# Digital Education of Health Professionals on the Management of Domestic Violence: Systematic Review and Meta-Analysis by the Digital Health Education Collaboration

**DOI:** 10.2196/13868

**Published:** 2019-05-23

**Authors:** Ushashree Divakar, Nuraini Nazeha, Pawel Posadzki, Krister Jarbrink, Ram Bajpai, Andy Hau Yan Ho, James Campbell, Gene Feder, Josip Car

**Affiliations:** 1 Centre for Population Health Sciences Lee Kong Chian School of Medicine Nanyang Technological University Singapore Singapore Singapore; 2 Psychology Programme School of Social Sciences Nanyang Technological University Singapore Singapore Singapore; 3 Palliative Care Centre for Excellence in Research and Education Singapore Singapore; 4 Department of Health Workforce World Health Organization Geneva Switzerland; 5 School of Social and Community Medicine University of Bristol Bristol United Kingdom

**Keywords:** systematic reviews, evidence-based, health workforce, domestic violence

## Abstract

**Background:**

The World Health Organization states that 35% of women experience domestic violence at least once during their lifetimes. However, approximately 80% of health professionals have never received any training on management of this major public health concern.

**Objective:**

The objective of this study was to evaluate the effectiveness of health professions digital education on domestic violence compared to that of traditional ways or no intervention.

**Methods:**

Seven electronic databases were searched for randomized controlled trials from January 1990 to August 2017. The Cochrane Handbook guideline was followed, and studies reporting the use of digital education interventions to educate health professionals on domestic violence management were included.

**Results:**

Six studies with 631 participants met our inclusion criteria. Meta-analysis of 5 studies showed that as compared to control conditions, digital education may improve knowledge (510 participants and 5 studies; standardized mean difference [SMD] 0.67, 95% CI 0.38-0.95; I^2^=59%; low certainty evidence), attitudes (339 participants and 3 studies; SMD 0.67, 95% CI 0.25-1.09; I^2^=68%; low certainty evidence), and self-efficacy (174 participants and 3 studies; SMD 0.47, 95% CI 0.16-0.77; I^2^=0%; moderate certainty evidence).

**Conclusions:**

Evidence of the effectiveness of digital education on health professionals’ understanding of domestic violence is promising. However, the certainty of the evidence is predominantly low and merits further research. Given the opportunity of scaled transformative digital education, both further research and implementation within an evaluative context should be prioritized.

## Introduction

Domestic violence (also referred to as family violence) is a complex public health problem [1] that places a notable burden on the health care system [2]. The World Health Organization (WHO) defined domestic violence, an aggressive and oppressive form of interpersonal violence, as a situation where an individual uses control tactics to emotionally, physically, sexually, or economically abuse a family member or past/current romantic partner [3]. Forms of control behavior can include but are not limited to psychological, physical, sexual, financial, and emotional abuse [4].

The WHO commissioned a multinational study on domestic violence with data collected from 10 countries, which showed that 13%-61% of women between the ages of 15-49 years had experienced physical abuse from their intimate partners at least once in their lifetime [3]. However, due to various reasons including shame, embarrassment, social stigma, and fear of and dependency on the abuser, survivors are often unwilling to reveal their difficulties to others [5,6]. Domestic violence can have both short- and long-term effects on the mental and physical well-being of the survivors. Injuries and physical ailments resulting from prolonged exposure to domestic violence include chronic neurological disorders, cardiovascular diseases, respiratory, intestinal and digestive conditions, reproductive disorders, physical injuries, and even death [7-10]. The less visible, but equally detrimental, impact of domestic violence includes psychological and emotional sufferings, anxiety, fear, depression, and posttraumatic stress [11]. Survivors often require treatment and care from a spectrum of health professionals, ranging from family physicians to physical therapists or clinical psychologists [5].

By facilitating early detection and treatment through a well-structured system of education and support, health professionals could play an important role in promoting greater awareness on domestic violence, identifying survivors of violence, and enabling survivor protection [12]. As health professionals are usually the first line of contact for survivors, they would need to undergo special training to identify, support, and treat domestic violence patients. This would be of particular importance to conservative societies where domestic violence is known to be severely underreported [13,14]. Moreover, the immense shortage of health professionals worldwide, especially in developing countries [15], compounded by the lack of training of health professionals, poses immense challenges in tackling the global domestic violence crisis [13,16].

Occasional training programs on domestic violence consisting of seminars and workshops often claim poor retention rates, as they are mostly time-consuming [17], require health professionals to travel to training locations, and are taught by academics who themselves may have had little exposure to people who have experienced domestic violence [18,19]. With the increasing use of information communication technologies in health professions’ education, leveraging on digital education to provide domestic violence management education could help address the various challenges of training and manpower shortage while improving the cost-effectiveness of educational programs [20-22].

The term digital education refers to a range of teaching and learning strategies that utilize digital media and devices for training and as interaction tools [23]. Digital education can be further subclassified into various types according to delivery methods (online or offline), content, learning objectives, pedagogical approaches, and delivery settings [24]. The use of the internet to deliver content is referred to as the online mode of digital education, while the use of software or PowerPoint without the need for the internet to deliver content is referred to as the offline mode of digital education. With its scalability, flexibility, cost-effectiveness, and ability to overcome geographical and temporal constraints, digital education has the potential to provide more independent, customized, and accessible domestic violence training. Studies comparing digital education to traditional methods in various specialties including medical education and engineering have found digital education to be more efficient and effective in building knowledge [25,26].

Although a previous study reviewed domestic violence education among health professionals [19], digital education has been gaining popularity in health professions’ curricula and hence its use in domestic violence education should be studied further. To the best of our knowledge, there are no systematic reviews evaluating the effectiveness of digital health interventions specifically for domestic violence training among health professionals. Hence, the objective of this study was to evaluate the effectiveness of health professions’ digital education on domestic violence compared to that of traditional ways or no intervention.

## Methods

### Search Strategy

We followed the Cochrane Handbook guidelines for this review. A more detailed description of the methodology is provided in the paper by Car et al [27]. This review is part of a global evidence-synthesis initiative for digital health professions’ education [28-39]. The search for the relevant trials was conducted across 7 databases: MEDLINE (Ovid), EMBASE (Elsevier), the Cochrane Central Register of Controlled Trials (Wiley), PsychINFO (Ovid), Educational Resource Information Centre (Ovid), Cumulative Index to Nursing and Allied Health Literature (EBSCO), and Web of Science Core Collection (Thomson Reuters). The detailed search strategy for MEDLINE is presented in the Multimedia Appendix 1. A manual search was conducted to identify any relevant articles from the reference lists of all included articles. A search was also conducted in the International Clinical Trials Registry Platform Search Portal and the metaRegister of Controlled Trials to identify unpublished trials, meeting abstracts, and doctoral theses from Jan 1990 to August 2017.

### Eligibility Criteria

The inclusion criteria are presented in Textbox 1. We adopted a broad definition of domestic violence, encompassing all subcategories of domestic violence, to capture a wide range of studies on the topic.

**Inclusion criteria for studies.**

Design

- Randomized controlled trials
- Cluster randomized controlled trials

Participants

- Preregistration undergraduates enrolled in health-related courses (including allied health, nursing, and rehabilitation specialization).
  - Preregistration undergraduate education or basic vocational training is defined as any type of study leading to a qualification that (1) is recognized by the relevant government or professional bodies of the country where the study was conducted and (2) entitles the qualification holder to apply for entry-level positions in the health care workforce or have direct contact with patients
- Postregistration health professionals undertaking Continued Medical Education and Continued Professional Development.
  - Postregistration is defined as any type of qualification that is recognized by the relevant government bodies and enables the holder to gain entry into or continue to work in the health care workforce in a more independent or senior role, excluding traditional/complementary medicine practitioners
  - Continued Medical Education is defined as “educational activities which serve to maintain, develop, or increase the knowledge, skills, and professional performance and relationships that a health professional uses to provide services for patients, the public, or the profession” [41]
  - Continued Professional Development is defined as “a range of learning activities through which health and care professionals maintain and develop throughout their career to ensure that they retain their capacity to practice safely, effectively and legally within their evolving scope of practice” [42]

Interventions/exposure

- Studies that use digital education interventions to train pre- and postregistration health professionals in domestic violence management
- Training is delivered via digital education alone (fully) or partially (ie, blended learning)

Comparator(s)/control

- Studies comparing digital education interventions with traditional methods of learning domestic violence management
- Studies comparing digital education interventions with control groups that do not receive any training on domestic violence management
- Studies comparing one type of digital education intervention to another

Outcomes

- Primary outcomes (assessed using validated or nonvalidated measurement tools):
  - Learners’ knowledge postintervention
  - Learners’ skills postintervention
  - Learners’ attitudes
  - Learners’ improvement of self-efficacy defined as improved efficiency toward domestic violence management
- Secondary outcomes (assessed using validated or nonvalidated measurement tools):
  - Learners’ satisfaction postintervention
  - Patient-related outcomes
  - Cost and cost-effectiveness of the intervention
  - Any adverse or unintended effects of digital education interventions

Timeline

- Publications from January 1990 through August 2017

We included randomized controlled trials (RCTs) and quasi-randomized trials reporting the use of digital education interventions (including blended learning, which is a combination of conventional learning and digital education) to educate health professionals on domestic violence management. RCTs with and without control groups that received traditional interventions delivered by either health professionals or university personnel were included. Studies targeting both practicing health professionals and students were included in this review. No language restrictions were imposed. All digital education interventions were included. Cross-over studies were excluded due to the high likelihood of carry-over effects [40]. Non-RCTs and studies not focusing on computer-based interventions and interventions delivered to individuals other than health professionals were also excluded.

### Study Selection

The search results from all the databases were combined in a single Endnote X8 library (Clarivate Analytics, Philadelphia, PA), and all duplicate records were removed. Search filters were used to remove articles not related to digital education for health professionals. Two reviewers (UD and NN) then independently screened the titles, abstracts, and full-text articles to identify studies potentially meeting the inclusion criteria. Disagreements were resolved through discussion between the reviewers. Primary outcomes included knowledge, skills, attitudes, self-efficacy, and satisfaction with the education measured using any validated and nonvalidated instruments. Secondary outcome measures included patient outcomes (eg, feedback from domestic violence survivors seeking treatment), change in health professionals’ behavior (ie, health professionals’ confidence in and ease of identifying and treating domestic violence survivors), and economic impact of the intervention.

### Data Extraction

All the relevant data including study characteristics, type of digital education intervention, participant demographics, data for outcome measures, and other publication details were extracted independently by UD and NN using a structured data extraction form. We contacted one study author (Short LM) [43] for missing information.

### Risk of Bias Assessment and the Overall Quality of Evidence

UD and NN independently assessed the risk of bias using the Cochrane Collaboration’s risk of bias tool [44]. When it was unclear if a trial was of low or high risk, the field was coded as unclear risk of bias. The following domains were evaluated: random sequence generation, allocation concealment, blinding of participants and personnel, blinding of outcome assessors, incomplete outcome data, selective outcome reporting, and other bias. The following GRADE (Grading of Recommendations Assessment, Development and Evaluation) criteria for evaluating the overall quality of evidence were used: limitations of studies (risk of bias), inconsistency (heterogeneity), indirectness, imprecision, and publication bias [45].

### Data Synthesis

Postintervention mean and SDs were used. The baseline mean value was used to calculate the final posttest mean and SD in studies that presented change scores rather than the final mean. When the studies compared more than two groups, the results from the comparison of the least active control group and the most active intervention group were presented.

### Statistical Analysis

We pooled the data using the random-effect model and calculated standardized mean differences (SMDs) with 95% CIs. Statistical heterogeneity across studies was assessed using the Cochran Q test and I^2^ statistics (negligible: 0%-40%, moderate: 30%-60%, or substantial: 50%-90% heterogeneity) [44]. All statistical analyses were conducted using RevMan software (version 5.3; The Nordic Cochrane Centre, Copenhagen, Denmark).

## Results

**Figure 1:**
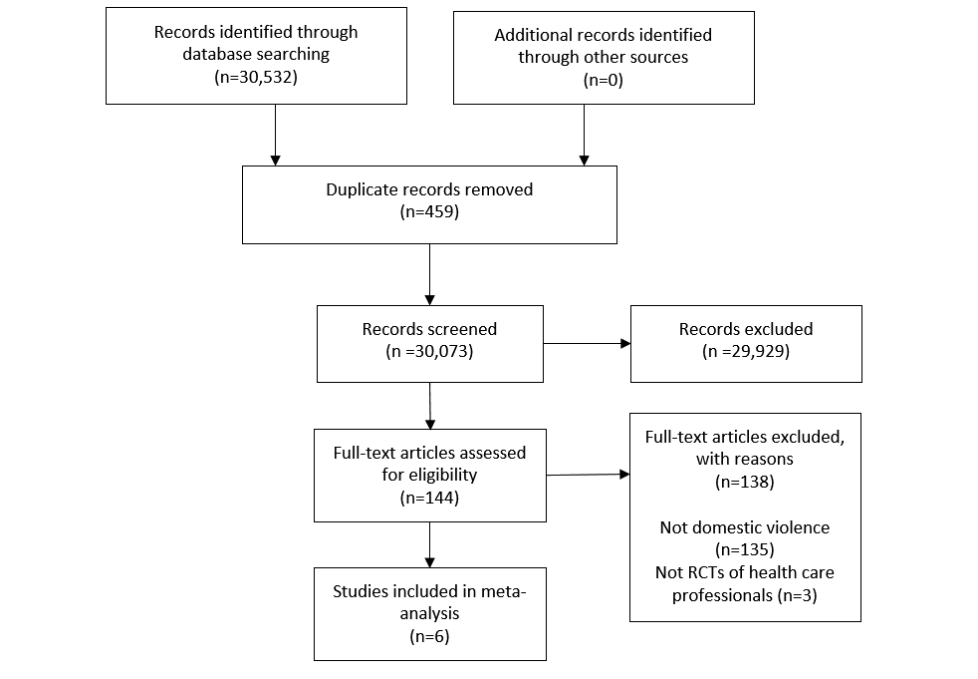
Preferred Reported Items for Systematic Reviews and Meta-analyses chart summarizing the selection process. RCT: randomized controlled trial.

The searches generated a total of 30,073 references. Following abstract and title screening, 144 articles were found to be relevant to domestic violence and selected for full-text screening. Of those, six met our eligibility criteria (Figure 1).

### Study Characteristics

All the 6 included RCTs were published between 2000 and 2014 in high-income countries: 5 were from the United States [43,46-49] and 1 was from the Netherlands [50]. All the studies included were parallel RCTs. Three of the studies were conducted in a university setting, and the remaining three were conducted in community and hospital settings. Two of the studies [49,50] focused on child abuse; one, on intimate partner violence [43], and three, on domestic violence, in general [46-48]. In addition, three studies targeted dental professionals [46,48,49], two targeted physicians [43,47], and one targeted nurses [50].

A total of 631 participants were included in the six studies, of which 420 participants (66%) were dentists and dental students. Three studies used offline and three studies used online modes of delivering digital education intervention. The following primary outcomes were reported: knowledge [43,46-49], attitudes [43,46-48], self-efficacy [43,47,50], and skills [50]. Surveys, questionnaires, and checklist were used to measure these outcomes, of which only two instruments [43,47] were validated. The duration of the intervention varied between 15 minutes and 3 weeks. Tables 1 and 2 summarize the main characteristics of the included studies. The study by Shapiro [49], including second-year dental students, on recognizing child abuse was the only study comparing a digital education intervention with traditional lecture-based learning, whereas all the other studies compared digital education to no intervention.

**Table 1:** Characteristics of the included studies.

Study (year), country, setting	Characteristics of participants (preregistration/postregistration/mixed) and field of study (number of participants)	Intervention (duration)	Control	Results
Danley et al (2004), USA, university [46]	Mixed (dental students and dentists); dentistry (N=174)	Offline interactive multimedia tutorial on DV^a^ designed to educate dentists to identify and respond to DV. Control group had no intervention. Assessment via questionnaires (15-25 min)	No intervention	Intervention demonstrated significantly improved attitudes and knowledge compared to the control group.
Harris et al (2002), USA, medical association [47]	Postregistration (physicians); primary care, emergency medicine, and orthopedics (N=121)	Online DV program designed to improve the confidence of practicing physicians in managing DV patients. Assessment via questionnaires (2 weeks to complete the program)	No intervention	Online education program on DV can improve physician confidence (measured by self-efficacy), attitudes, and self-reported knowledge in managing DV patients. In addition, 17.8% mean change in the self-efficacy domain score for the intervention group versus –0.6% change for the control group (*P*<.001) was observed. Self-reported user satisfaction with the program was high.
Hsieh et al (2006), USA, university and clinics [48]	Postregistration (dentists); dentistry (N=174)	Offline interactive multimedia tutorial on DV designed to educate dentists to identify and respond to DV. Assessment via questionnaires (15 min)	No intervention	The posttest comparison of the two groups was statistically significant (*P*=.01) in favor of the online training group.
Shapiro et al (2014), USA, university [49]	Preregistration (dental students); dentistry (N=72)	Online interactive training module to educate dental students on child abuse, assessed via questionnaires (3 weeks for reviewing the online module)	Traditional lecture-based session	In LG^b^, 91.6% agreed or strongly agreed that the traditional lecture was a good way to learn the material.
Short et al (2006), USA, community practice [43]	Postregistration (community physicians); family medicine, pediatrics, obstetrics, and gynecology (N=52)	Online CME^c^ program to educate HCPs^d^ on IPV^e^ program in a community practice setting assessed via self-administered, paper-based survey tool (minimum 4 hours)	No intervention	Online CME^f^ survey program for physician readiness to manage intimate partner violence was successful in improving physicians’ IPV knowledge, attitudes, and self-efficacy.
Smeekens et al (2011), The Netherlands, medical center [50]	Postregistration (nurses); emergency medicine (N=38)	Offline program designed to educate nurses to recognize child abuse in a simulated case, assessed via performance in simulated cases (minimum of 2 hours during a 2-week period)	No intervention	Nurses in the intervention group performed significantly better during the simulation than the control group and reported higher self-efficacy.

^a^DV: domestic violence.

^b^LG: lecture group

^c^CME: Continued Medical Education.

^d^HCP: health care professional.

^e^IPV: intimate partner violence.

^f^Continued Medical Education is defined as “educational activities which serve to maintain, develop, or increase the knowledge, skills, and professional performance and relationships that a health professional uses to provide services for patients, the public, or the profession” [48].

**Table 2:** Outcomes of the included studies.

Study and outcome measures		Intervention group score, mean (SD)	Control group score, mean (SD)
**Danley et al** [46]			
	Knowledge	3.0 (0.76)	2.1 (0.78)
	Attitude	4.6 (1.15)	3.9 (1.08)
**Harris et al** [47]			
	Knowledge	3.3 (1.96)	2.5 (0.02)

	Attitude	—^a^	—
	Satisfaction	—	—
	Self-efficacy	3.7 (1.20)	3.3 (0.04)
**Hsieh et al** [48]			
	Knowledge	3.1 (2.29)	2.3 (0.18)
	Attitude	5.5 (0.19)	4.8 (1.25)
**Shapiro et al** [49]			
	Knowledge	80.5 (1.24)	76.1 (1.56)
	Satisfaction	—	—
**Short et al** [43]			
	Knowledge	28.4 (5.68)	25.8 (5.68)

	Attitude	4.7 (1.00)	3.5 (1.00)
	Self-efficacy	4.6 (1.15)	3.8 (1.15)
**Smeekens et al** [50]			
	Skills	71 (18)	89 (19)

	Self-efficacy	447 (98)	502 (96)

^a^Not available.

### Effects of Interventions

**Figure 2:**
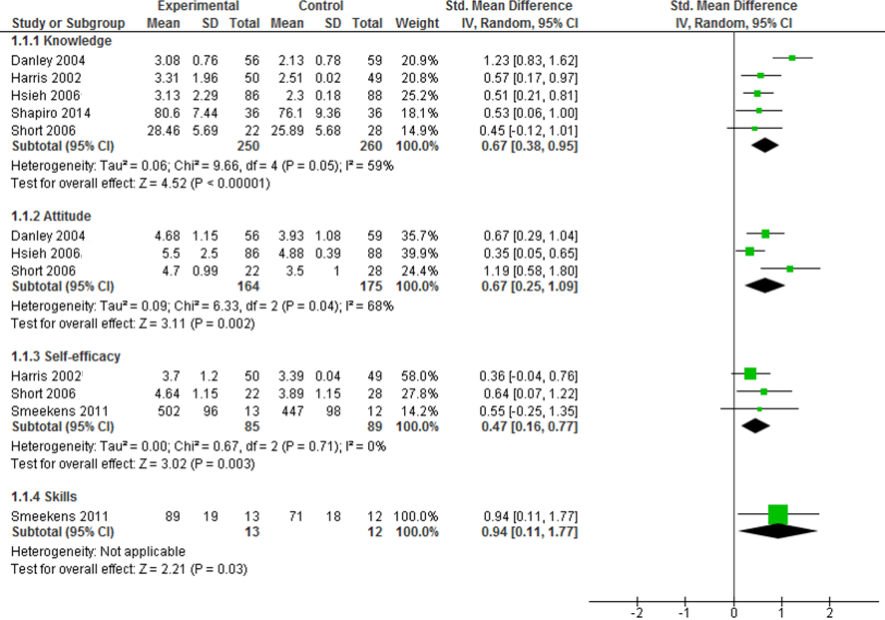
Forest plot comparing the experimental and control groups in terms of outcomes. IV: interval variable; random: random effect model; std: standardized.

Meta-analysis of five studies [43,46-49] considered to be sufficiently homogeneous found that digital education (offline and online) may increase knowledge of domestic violence in dentists, physicians, and allied health professionals (510 participants; SMD 0.67, 95% CI 0.38-0.95; I^2^=59%; low certainty evidence) compared with no intervention and traditional learning postintervention. There was evidence of moderate heterogeneity among the studies (ζ²=0.06; χ²_4_=9.7; *P*=.05; I²=59%; Figure 2).

Meta-analysis of three studies [43,46,48] found that compared to no intervention, digital education (offline and online) may increase postintervention attitude toward domestic violence management in dentists and physicians (339 participants; SMD 0.67, 95% CI 0.25-1.09; I^2^=68%). There was a substantial level of heterogeneity among the studies (ζ²=0.09; χ²_2_=6.3; *P*=.04; I²=68%).

Meta-analysis of three studies [43,47,50] found that compared to no intervention, digital education (offline and online) may increase postintervention self-efficacy toward domestic violence management in physicians and nurses (174 participants; SMD 0.47, 95% CI 0.16-0.77; I^2^=0%). The was no evidence of heterogeneity (ζ²=0.00; χ²_2_=0.7; *P*=.71; I²=0%).

One study [50] comparing change of score in skills found that digital education (offline program) may improve domestic violence skills in nurses (25 participants; SMD 0.94, 95% CI 0.11-1.77) compared to no intervention.

### Summary Risk of Bias

Of the six studies, four [43,46,48,50] were found to have an overall low risk of bias and the remaining two [47,49] had a high or an unclear risk of bias.

**Figure 3:**
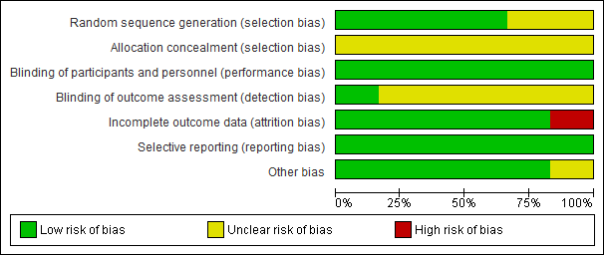
Risk of bias summary.

The random sequence generation method was reported in four [43,46,48,50] of the six studies. Blinding and protection against selective reporting was achieved through the nature of the intervention and the reporting of all the results in all the studies. Attrition and other biases were of low risk for five [43,46,48-50] of the six studies. One study had a high risk of attrition bias resulting from a high drop-out rate (42%). However, details of allocation concealment were not reported in any of the studies, and blinding of outcome assessment was attempted in only one study [50]. Similarly, the method for random sequence generation was not clearly stated in two studies [47,49]. At the individual-study level, of 56 domains, 14 (25%) were reported as unclear and one (2%) was reported as high risk (Figure 3). The summary of findings table shows the evidence to be of low to moderate quality as analyzed per the GRADE criteria (Table 3).

**Table 3:** Summary of findings table. Patient or population: health care professionals; Setting: university; Intervention: digital education; Comparison: traditional or no intervention.

Outcomes	Anticipated absolute effects^a^ (95% CI)		Number of participants (number of RCTs^b^)	Certainty of the evidence (GRADE^c^)	Comments
	Assumed risk with controls	Corresponding risk with electronic learning			
Knowledge	The mean outcome score in the control groups was 21.79	The mean knowledge score in the intervention groups was 0.67 SD higher (0.38-0.95 higher)	510 (5)	Low^d,e,f^	None
Attitude	The mean outcome score in the control groups was 4.10	The mean attitude score in the intervention groups was 0.67 SD higher (0.25-1.09 higher)	339 (3)	Low^d,e,f^	The results of one study (121 participants) were not pooled due to incomplete data
Self-efficacy	The mean outcome score in the control groups was 151.43	The mean self-efficacy score in the intervention groups was 0.47 SD higher (0.16-0.77 higher)	174 (3)	Moderate^e,g^	None
Skills	The mean outcome score in the control groups was 71	The mean skill score in the intervention groups was 0.94 SD higher (0.11-1.77 higher)	25 (1)	Low^d,e,f^	None

^a^The risk in the intervention group (and its 95% CI) is based on the assumed risk in the comparison group and the relative effect of the intervention (and its 95% CI).

^b^RCT: randomized controlled trial.

^c^GRADE: Grading of Recommendations Assessment, Development and Evaluation.

^d^The heterogeneity was high with large variations in effects and the lack of overlap among CIs.

^e^Rated down by one level for study limitations. The risk of bias was unclear for allocation concealment in all studies.

^f^Low: Our confidence in the effect estimate is limited. The true effect may be substantially different from the estimate of the effect.

^g^Moderate: We are moderately confident in the effect estimate. The true effect is likely to be close to the estimate of the effect, but there is a possibility that it is substantially different.

## Discussion

In this paper, we systematically reviewed and pooled data on the use of digital education for domestic violence management. We draw attention to the gap in digital education on domestic violence and the potential benefits of this educational strategy. Our findings provide preliminary evidence to show that using digital education to address socially sensitive issues such as domestic violence may improve certain educational outcomes in health professionals receiving the training. Although competencies, trustable professional activities, knowledge, skills, and attitudes do not automatically translate into change of practice, they are indispensable for improving patient outcomes.

All the studies included in this review were published after the year 2000, which is the period when digital education started becoming more prominent in health care professions’ education [51] and more laws were implemented to tackle domestic violence [5,52,53].

Interestingly, we found that in all the studies, the intervention groups had improved knowledge, skills, attitudes, and self-efficacy, even though the studies employed different methodologies, sample sizes, sampling periods, settings, and types of domestic violence education. Additionally, the changes in primary outcomes were observed within short time periods of up to 2 weeks after the intervention, with only one study [43] measuring retention at the 12-month follow-up. Although the variability suggests that digital education has the versatility to reach a wide range of health professionals in different populations and settings, it underscores the potential of homogeneous short-term digital interventions in improving the quality of care that these professionals provide.

Risk of bias was mostly unclear for blinding of outcome assessment and allocation concealment, but it was mainly low for sequence generation. While the nature of the interventions does not allow blinding of participants, we believe it would not have had any effect on bias risk. We minimized biases by having two reviewers independently assess the articles for inclusion, complete data extraction, risk of bias, and use of the GRADE criteria. The overall quality of the evidence was low or moderate due to the risk of bias and inconsistency across the studies (Table 3).

This review has some important strengths including a strict adherence to the gold-standard Cochrane methods and use of validated, comprehensive, and reproducible searches across seven databases. Our review adds to previous research on domestic violence education for health professionals, as it focuses on the use of digital education, which is a growing area of research. Some weaknesses have to be kept in mind when interpreting the results of this systematic review. For instance, although our searches were comprehensive, we cannot be certain that all relevant trials were included.

However, the evidence evaluated has some limitations. First, only a few studies were published in this area, and they were all from high-income countries, making generalizability challenging. Although digital education may potentially serve as an effective and impactful solution to educating health professionals in domestic violence management, applicability, scalability, and implementation in low- and middle-income countries have to be studied further [54]. Second, we acknowledge that in certain countries such as the United Kingdom, social workers are the first “line of response” to domestic violence. Third, only two studies [47,49] measured and reported learners’ satisfaction as one of the primary outcomes. This further highlights the need for uniform and validated outcomes and methods of measuring them to make conclusive judgements. Moreover, only two studies [43,47] used validated measurement instruments to measure outcomes, thereby making it challenging to compare the use of digital education between settings. Subsequently, the lack of data on retention rate, costs, or patient outcomes prevents policy makers from making informed decisions or assessing the transferability of digital education to other settings. Finally, none of the RCTs reported secondary outcomes such as patient outcomes, health professionals’ behavior change, and economic impact. Hence, we are unable to assess how these outcomes changed with digital education.

Future studies should be designed to evaluate the effect of digital education on these outcomes. We further recommend that future studies consider including other professionals such as social carers, psychologists, counsellors, or teachers. Findings of this review suggest that digital education could contribute to developing the competencies that health professionals need to respond to complex psychosocial problems such as domestic violence. Therefore, future studies should focus on recording more practical outcomes of the trainings such as change in detection and referral rates [55]. This will help ensure a better understanding of the actual value of integrating digital education modules into pre- and postregistration as well as the continuing professional development curricula. In addition, although domestic violence is more accepted and prevalent in low- and middle-income countries, the education gap is wide and digital education is still at the developing stage in these countries [14,56]. Data should be collected beyond geographical regions with inclusion of the cost analysis to obtain a better understanding of the impact and feasibility of integrating electronic learning modules on domestic violence management into the medical curriculum in low- and middle-income countries. We further recommend that future studies be designed with larger, appropriately powered RCTs, in both developed and low- and middle-income countries alike in order to ensure better representation. Researchers could use methods such as the Consolidated Standards of Reporting Trials for Social and Psychological Interventions 2018 (CONSORT-SPI 2018) checklist as guidance to design and report future studies on digital education for domestic violence in order to ensure that the data collected are of high quality and representative [57].

We believe that while digital education could help increase identification of and support to patients experiencing domestic violence, research with study designs incorporating blended learning might hold the highest potential. Such designs would combine the best of digital education, such as smartphones, apps, emails, text messages, and virtual patients, with the best of traditional classroom practices such as personalized contact or feedback, meetings, and discussions.

In conclusion, we found some promising, predominantly low-quality evidence for the effectiveness of digital education on domestic violence. We also highlighted the need for further research evaluating and validating culturally tailored digital education interventions geared toward more holistic management of domestic violence.

## Appendix

Multimedia Appendix 1 MEDLINE (Ovid) search strategy.

## Abbreviations

CME: Continued Medical Education
CONSORT-SPI: Consolidated Standards of Reporting Trials for Social and Psychological Interventions
DV: domestic violence
GRADE: Grading of Recommendations Assessment, Development and Evaluation
HCP: health care professional
IPV: intimate partner violence
LG: Lecture Group
RCT: randomized controlled trial
SMD: standardized mean difference
WHO: World Health Organization
